# Supplementary material for: Synergistic Amylase and Debranching Enzyme Catalysis to Improve the Stability of Oat Milk
Source: Foods. 2025 Apr 5;14(7):1271. doi: 10.3390/foods14071271 (PMC11988502; doi:10.3390/foods14071271)
Supplement: Supplementary file 1 [file foods-14-01271-s001.zip › foods-3537470-supplementary.pdf]

Supplementary material

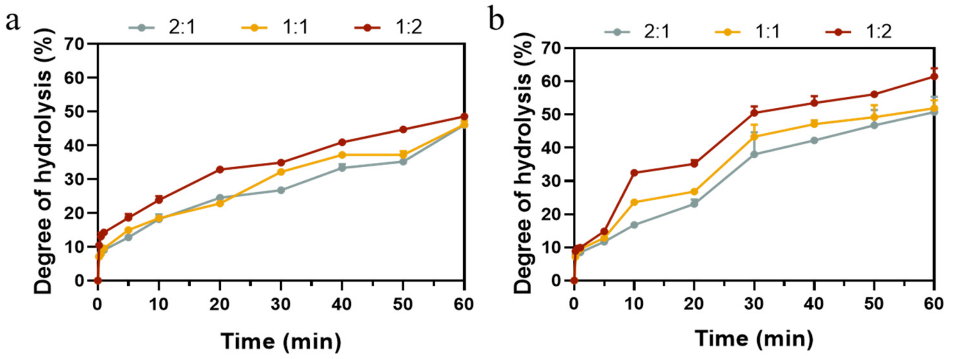

Figure S1. Effect of different ratios of pullulanase and isoamylase on the degree of hydrolysis of (a) oat milk and (b) OS. 2:1, treated with pullulanase and isoamylase at a ratio of 2:1. 1:1, treated with pullulanase and isoamylase at a ratio of 1:1. 1:2, treated with pullulanase and isoamylase at a ratio of 1:2.

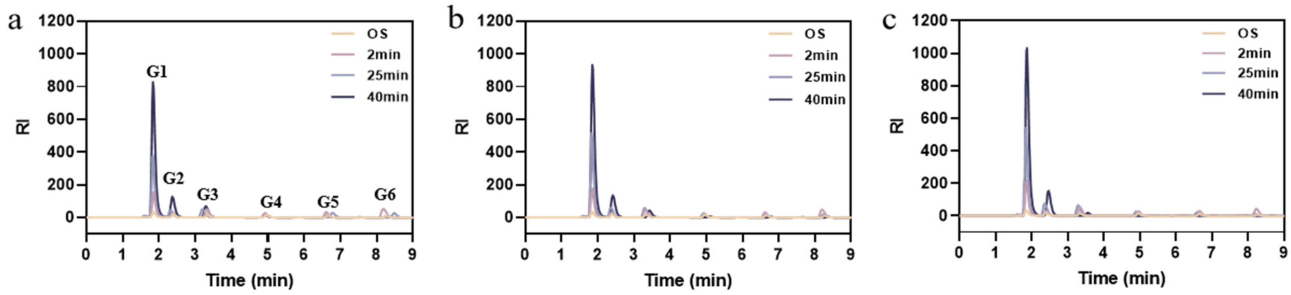

Figure S2. The composition of monosaccharides in the EHOS with (a) 2:1, (b) 1:1, and (c) 1:2 ratios of pullulanase and isoamylase. OS, oat starch; 2 min, treated for 2 min; 25 min, treated for 25 min; 40 min, treated for 40 min. G1, glucose; G2, maltose; G3, maltotriose; G4, maltotetraose; G5, maltopentose; G6, maltohexaose.

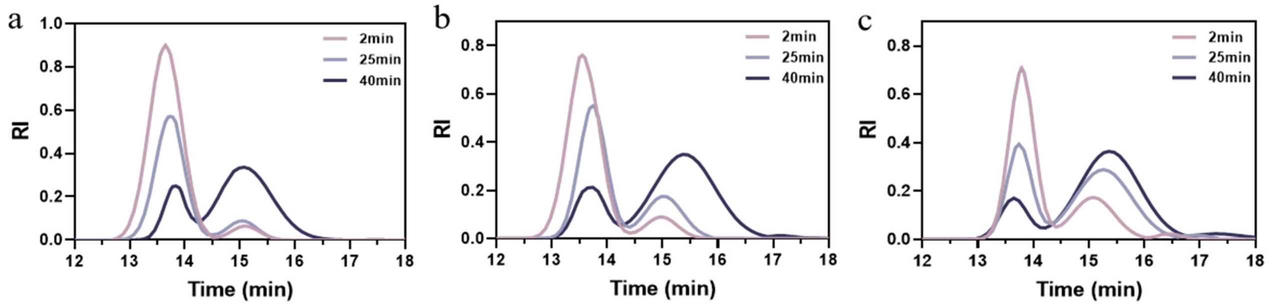

Figure S3 Chain length distributions of EHOS with (a) 2:1, (b) 1:1, and (c) 1:2 ratios of pullulanase and isoamylase. 2 min, treated for 2 min; 25 min, treated for 25 min; 40 min, treated for 40 min.

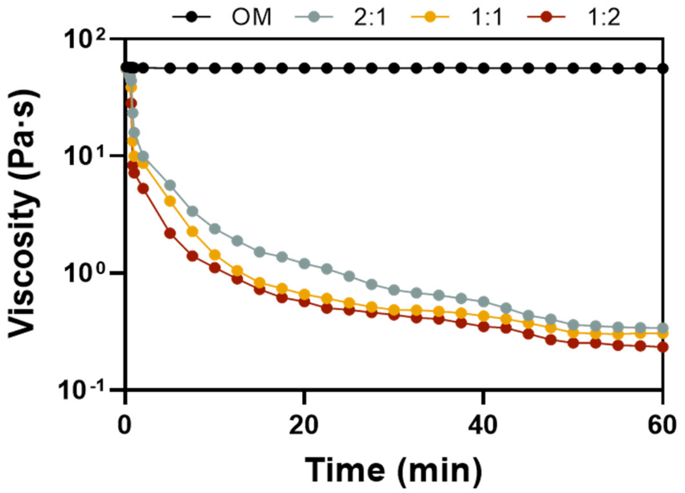

Figure S4. Viscosity curve of oat milk treated by different ratios of debranching enzyme. 2:1, treated with pullulanase and isoamylase at a ratio of 2:1. 1:1, treated with pullulanase and isoamylase at a ratio of 1:1. 1:2, treated with pullulanase and isoamylase at a ratio of 1:2.

Table S1. Molecular weight (Mw) and polydispersity index (PDI) of OS and EHOS.

	Peak 1		Peak 2		Peak 3		Peak 4	
	Mw (Da)	PDI	Mw (Da)	PDI	Mw (Da)	PDI	Mw (Da)	PDI
OS	1078797	13.65	-	-	-	-	213	1.07
$2PI_{2:1}$	-	-	45579	3.40	1537	1.76	217	1.05
$2PI_{1:1}$	-	-	39817	2.88	1550	1.69	226	1.07
$2PI_{1:2}$	-	-	29070	2.34	1467	1.59	234	1.06
$25PI_{2:1}$	908964	2.08	14834	2.27	1163	1.22	280	1.13
$25PI_{1:1}$	896765	2.23	12755	2.27	1107	1.17	290	1.16
$25PI_{1:2}$	836069	2.20	-	-	7324	3.20	342	1.26
$40PI_{2:1}$	-	-	-	-	5434	2.41	343	1.26
$40PI_{1:1}$	-	-	-	-	5212	2.37	336	1.26
$40PI_{1:2}$	299732	1.15	-	-	4802	2.26	321	1.24

Data are shown as mean ± standard error. Letters represent significant differences ($p < 0.05$). OS, oat starch; $2PI_{2:1}$/$2PI_{1:1}$/$2PI_{1:2}$, treated for 2 min at pullulanase to isoamylase ratios of 2:1, 1:1, and 1:2; $25PI_{2:1}$/$25PI_{1:1}$/$25PI_{1:2}$, treated for 25 min at pullulanase to isoamylase ratios of 2:1, 1:1, and 1:2; $40PI_{2:1}$/$40PI_{1:1}$/$40PI_{1:2}$, treated for 40 min at pullulanase to isoamylase ratios of 2:1, 1:1, and 1:2.
